# Supplementary material for: Understanding Alveolar echinococcosis patients’ psychosocial burden and coping strategies—A qualitative interview study
Source: PLoS Negl Trop Dis. 2023 Aug 4;17(8):e0011467. doi: 10.1371/journal.pntd.0011467 (PMC10403068; doi:10.1371/journal.pntd.0011467)
Supplement: Attachment — Submitted filename: cover letter_echino_qual_revision_nikendei et al..docx [file pntd.0011467.s001.docx]

**PLOS Neglected Tropical Diseases**

Section Editor: Francesca Tamarozzi

**Submission date: 23.04.2023**

**Revised manuscript "Understanding Alveolar Echinococcosis Patients' Psychosocial Burden and Coping Strategies - A Qualitative Interview Study" (#PNTD-D-23-00092) for publication in PLOS Neglected Tropical Diseases.**

Dear Section Editor Francesca Tamarozzi,

Thank you very much for your email. We greatly appreciate the critical and helpful points raised by the section editor and the two reviewers. The comments and suggestions were very helpful in substantially improving the manuscript and we have carefully revised the manuscript according to the recommendations. In this cover letter, we have explained all changes made to the manuscript in detail and have provided exact references regarding their location in the revised version of the manuscript. Furthermore, all changes were marked in the text. We would like to express our gratitude for giving us the opportunity of submitting a revised version of Manuscript No. # PNTD-D-23-00092.

Editorial comment:

Dear Prof. Dr. Nikendei

Thank you very much for submitting your manuscript "Understanding Alveolar Echinococcosis Patients' Psychosocial Burden and Coping Strategies - A Qualitative Interview Study" for consideration at PLOS Neglected Tropical Diseases.

As with all papers reviewed by the journal, your manuscript was reviewed by members of the editorial board and by several independent reviewers. The reviewers appreciated the attention to an important topic. Based on the reviews, we are likely to accept this manuscript for publication, providing that you modify the manuscript according to the review recommendations.

Thank you again for your submission to our journal. We hope that our editorial process has been constructive so far, and we welcome your feedback at any time. Please don't hesitate to contact us if you have any questions or comments.

Sincerely,

Francesca Tamarozzi

Section Editor

PLOS Neglected Tropical Diseases

Response to editorial comment:

Dear Francesca Tamarozzi,

Thank you very much for these encouraging editorial comments. We are most delighted about this feedback and the editorial process. Please find our detailed answers below, as well as the revised version of the manuscript uploaded in the manuscript central.

***********************

Reviewer`s comments:

**Key Review Criteria Required for Acceptance**

Reviewer #1:

The manuscript is well written. The objectives of the study and the population are clearly described. The design seems appropriate and the sample size sufficient from the viewpoint of a clinician involved in the care of AE patients, but this should also be reviewed by an expert in the field of psychological research. I see no concerns regarding ethical or regulatory requirements.

Response to comment of Reviewer #1:

Dear Reviewer #1,

Thank you very much for your general comment and feedback. We also appreciated your critical comments that we aimed to thoroughly address below.

Reviewer #2:

The objectives of the study are clearly articulated. The study design is fairly straightforward. More rational should be provided about the chosen method (semi-structured interviews)- why was this appropriate for conduct a study of this kind? Further information should also be provided in the limitations of this method for understanding psychological wellbeing and how conducting interviews in a hospital setting may have shaped responses- this is not appropriately addressed currently. There are also some leading questions 'do you feel stigmatised' in the topic guides (table 1). More discussion of this should be provided and again this should be identified as a limitation.

Response to comment of Reviewer #2:

Dear Reviewer #2,

Thank you very much for your detailed response and valuable comments on the manuscript. It is absolutely correct that qualitative semi-structured interviews represent only one of the methods available in terms of qualitative research. Interviews following a grounded theory approach or focus groups represent further important methodological approaches in qualitative research. As we were interested in specific in-depth insights regarding mental health of AE patients and the fact that patients could only be included in the study during their individual clinical appointments, we chose semi-structured interviews as a suitable methodological approach. Qualitative interviews serve the purpose of obtaining in-depth information and insights that go beyond measures from standardized questionnaires. Here, half-standardised interviews seem adequate and helpful as they leave room with open questions, followed by encouraging questions and specifying questions. Arguments for the qualitative approach chosen are now given in the methods section (please see page 6, paragraph 3):

“Due to the specific research questions and the approach of AE patients’ consecutive recruitment during routine AE check-ups, a methodological approach using half-standardized individual interviews was chosen.”

It is absolutely correct that a research study in the environment of clinical treatment might lead to biases, e.g. in terms of social desirability. This, indeed, cannot be ruled out, and the limitations section is now broadened accordingly (please see page 16, paragraph 2):

“This study has several limitations. First, as is customary in qualitative research, it relies on self-report interviews. Accordingly, we cannot rule out a social desirability bias. Such a bias might even be more prominent, as interviews were conducted in a clinical setting where AE patients seek help for medical monitoring and check-up.”

**Results**

Reviewer #1:

The analysis is mostly conclusive and clearly and completely presented. The tables are of sufficient quality and clarity.

Response to comment of Reviewer #1:

Thank you for this positive response regarding the results section.

Reviewer #2:

The analysis is clear, however, it is a little deductive, and i would have expected to see more interconnection between the themes presented. For example, there is a clear link between the information provided in A2 and B1 that needs to be discussed together so that it is easy to the reader to see the bigger picture in the diagnosis communication pathway and how this shapes psychological wellbeing. C1/C2 could also be strengthened by considering these as forms of stigma as opposed to the themes currently presented- engaging with the stigma literature would support this part of the analysis. Finally, there is limited consideration of how socio-demographics shape the responses- adding this throughout would strengthen the results.

Response to comment of Reviewer #2:

This represents a very important and valuable comment. We worked hard on improving the discussion and to establish the interconnection of paragraphs that is clearly needed (please see amendments highlighted throughout the discussion section):

“This directly points to psychosocial interventions that have to be provided and tailored to AE patient`s needs, which are discussed below.”

“Psychotherapeutic interventions have to address coping strategies, promotion of (pre-)existing key resources, the enhancement of self-efficacy, while recognizing and acknowledging somatic restrains, reflecting on interpersonal conflicts arising from psychological and somatic symptoms, and facilitating family and social environment support.”

“Such interventions are urgently needed, as psychosocial family and social environment support are highly needed and desired by AE patients, and at the same time, family and social environment seems to be a source of stigmatization that is most feared. Our data clearly indicates that a family approach is necessary, including AE-related information for reducing stigmatization tendencies, facilitation of psychosocial support, and the exploration of the patient's family system for establishing helpful interpersonal relationships.”

“These aspects have to be an indispensable part of psychotherapeutic topics and interventions, as outlined above.”

“These interpersonal processes have also to be addressed and accompanied during the psychotherapeutic process.”

Furthermore, we were happy to underpin observations regarding stigmatization with related literature that is now cited (please see page 15, paragraph 1):

“Stigmatization in patients suffering from infectious diseases is widely observed, can have an important impact on mental health [36], and contribute to a lower health-related quality of life [37]. Therefore, it is important to explore suitable interventions for the reduction of stigmatization if needed. In patients with chronic diseases illness, disclosure and use of coping strategies can be protective factors [38].”

It is absolutely correct that there is limited consideration of how socio-demographics shape the responses. However, considering the sample size, drawing conclusions from such a subgroup analysis seems improper. However, this comments points towards important future research topics, which is a fact now mentioned in the limitations section (please see page 16, paragraph 2):

“In order to learn more about differing socio-economic aspects and age on research topics such as internet use, further research is needed, as the presented sample size is too small to allow such subgroup orientated conclusions.”

**Conclusions**

Reviewer #1:

The conclusions are supported by the presented data and relevant limitations to the study discussed. There is a great public health relevance as the topic of psychosocial burden and coping strategies in the field of AE is understudied. The presented analysis well reflects clinical experience in the care of AE patients.

Response to comment of Reviewer #1:

Thank you for this validation of our conclusions from the perspective of a clinician working with AE patients.

Reviewer #2:

The conclusions are supported by the data presented and this is an important study. There are a few areas of the discussion that should be revisited, including the section on depression. Currently, this is written from a very medicalised perspective (e.g. provide antidepressants), with limited exploration of how the personal and environmental factors presented in the results could also be useful to consider in intervention development. Additionally, family intervention is described as important, yet these are also the main points of stigma identified in the results. This should be considered/elaborated within the discussion.

Response to comment of Reviewer #2:

This observation is absolutely correct. We have now broadened the perspective on different psychosocial and psychotherapeutic topics, as well as interventions that are potentially needed and have to be considered for patient support (please see page 13, paragraph 1):

“Psychotherapeutic interventions have to address coping strategies, promotion of (pre-)existing key resources, and the enhancement of self-efficacy, while recognizing and acknowledging somatic restrains, reflecting on interpersonal conflicts arising from psychological and somatic symptoms, and facilitating family and social environment support.”

Furthermore, in the discussion section, we now clearly indicate if the word medication stands for AE medication or psychopharmacological medication.

**Editorial and Data Presentation Modifications**

Reviewer #1:

Due to the small sample size, I suggest using Median and Range instead of Mean and Standard Deviation in Table 2. Furthermore, the "AE Status" classification in Table 2 into "critical", "uncritical" and "cured" is uncommon and not defined in the methods section. I suggest classifying patients into the categories differently, i.e. "surgically cured", "inoperable - no biliary or vascular complications" and "inoperable - with biliary or vascular complications".

Response to comment of Reviewer #1:

Median and Range are now used instead of Mean and Standard Deviation in Table 2 (see page 22). Furthermore, we are aware of the fact that our “AE Status” classification might be uncommon. However, this was chosen deliberately to underline the important group of patients who experience significant adverse benzimidazole effects, complications, or even show progression under benzimidazole medication. These patients are highlighted in the category “critical”. In our clinical practise, their treatment is often challenging. We think that in a different classification, like "surgically cured", "inoperable - no biliary or vascular complications", and "inoperable - with biliary or vascular complications", this patient group is not sufficiently taken into account. Therefore, we would prefer to retain our original classification. Additionally, we defined our classification in the methods section (please see page 6, paragraph 2):

“Regarding AE status, patients were classified into the categories ‘critical’, ‘uncritical’, and ‘cured’. The following AE patients are defined as ‘critical’: AE lesion(s) unresectable, AE progression not well controlled by benzimidazoles, significant adverse benzimidazole effects and/or complications, such as cholangitis, biliary system obstruction, compression of blood vessels or adjacent organs, or distant metastases. ‘Uncritical’ contains unresectable AE lesion(s), but AE progression well controlled by benzimidazoles. ‘Cured’ are patients free of disease at long term follow-up, after resection of AE lesion(s) and two years of postoperative benzimidazoles.”

Reviewer #2:

(No Response)

Response to comment of Reviewer #2:

-

**Summary and General Comments**

Reviewer #1:

As mentioned above the study is of high academic and clinical relevance and the manuscript is well-written.

Response to comment of Reviewer #1:

Thank you again for your encouraging feedback.

Reviewer #2:

This paper is important and will make a good contribution to the literature once the issues outlined above are addressed.

Response to comment of Reviewer #2:

Thank you very much for your critical comments and the positive overall feedback.

***********************

Technical check results provided by Nithya Venkatesan:

Dear Dr Nikendei,

Thank you for submitting your paper, "Understanding Alveolar Echinococcosis Patients' Psychosocial Burden and Coping Strategies - A Qualitative Interview Study", to PLOS Neglected Tropical Diseases. We have checked over your submission and, before we can assign your manuscript to the Editor, we ask that you first please make the following modifications:

1) Thank you for stating "Data cannot be shared publicly because the interview transcripts contain personal data including information that may allow personal identification of participants". Please include the contact information for the third party organization that should be contacted should other researchers want to request access to this data and please include the full citation of where the data can be found. We also request that you verify with us via email that any researcher will be able to obtain the data set in the same manner that the you have obtained it. If you feel you are unwilling or unable to adhere to this policy, please explain your reasons by return email and your exemption request will be escalated to the editor for approval. Your exemption request will be handled independently and will not hold up the peer review process, but will need to be resolved should your manuscript be accepted for publication. One of the Editorial team will be in touch if they require more information.

Response to comment 1:

Dear Nithya Venkatesan, thank you for the technical check regarding our manuscript "Understanding Alveolar Echinococcosis Patients' Psychosocial Burden and Coping Strategies - A Qualitative Interview Study”. We took your requests most seriously. We fully support sharing of data upon reasonable request. The request can be submitted to the ethic committee of the University of Heidelberg. This fact is now stated in the methods section (see page 5, paragraph 3):

“Request for access to data can be addressed to ethikkommission-I@med.uni-heidelberg.de (ethics committee of the University of Heidelberg).”

2) Please provide an Author Summary. This should appear in your manuscript between the Abstract (if applicable) and the Introduction, and should be 150–200 words long. The aim should be to make your findings accessible to a wide audience that includes both scientists and non-scientists. Sample summaries can be found on our website under Submission Guidelines: [https://journals.plos.org/plosntds/s/submission-guidelines#loc-parts-of-a-submission](https://journals.plos.org/plosntds/s/submission-guidelines#loc-parts-of-a-submission)

Response to comment 2:

The author summary is now provided on page 3:

“Alveolar echinococcosis (AE) is a serious parasitic zoonotic disease that resembles malignancy with clinically silent infiltrative growth predominantly involving the liver. Semi-structured interviews were conducted with N = 12 AE patients focusing on their disease-related psychosocial burden, coping strategies, information seeking behavior, and subjective illness concepts. All participants perceived AE as a severe disease with inextricably linked biological, psychological, and social effects. Key positive influences reported included the provision of information and access to informal and formal support, including the ability to lead active personal and professional lives for as long as possible. Self-directed, web-based information seeking often led to increased feelings of hopelessness and anxiety. Disease-related apprehensions, and to prevent stigmatization, health professionals need to provide AE patients with comprehensive disease-related information to improve patient and social awareness.”

3) Thank you for including your ethics statement: "All participants gave informed consent and could withdraw their participation without disadvantage. All evaluated data were anonymized. The study was approved by the Heidelberg University Hospital Ethics Committee (Ethics Application No. S-232/2016). The study was conducted in accordance with the Declaration of Helsinki (most recent version: Fortaleza, Brazil, 2013)". Please state "whether written/verbal consent was obtained from the Participants".

Response to comment 3:

We now explain that for every patient verbal as well as written consent was obtained (see page 5, paragraph 4):

“All participants gave their verbal and written informed consent and could withdraw their participation without disadvantage. All evaluated data were anonymized. The study was approved by the Heidelberg University Hospital Ethics Committee (Ethics Application No. S-232/2016). The study was conducted in accordance with the Declaration of Helsinki (most recent version: Fortaleza, Brazil, 2013)”.

We are very grateful for the technical check performed, all the reviewers’ feedback, helpful comments, and their assistance to substantially improve our manuscript. All suggestions have been very valuable in revising the manuscript. We very much appreciate the time and effort that was invested into reviewing our manuscript and we hope that we have addressed all aspects sufficiently.

Thank you for giving us the opportunity to submit a revised version of PNTD-D-23-00092.

For the authors

Christoph Nikendei

Anastasiya Berkunova
